# Ratiometric control of cell phenotypes in monostrain microbial consortia

**DOI:** 10.1098/rsif.2022.0335

**Published:** 2022-07-13

**Authors:** Davide Salzano, Davide Fiore, Mario di Bernardo

**Affiliations:** ^1^ Department of Electrical Engineering and Information Technology, University of Naples Federico II, Via Claudio 21, 80125 Naples, Italy; ^2^ Department of Mathematics and Applications ‘R. Caccioppoli’, University of Naples Federico II, Via Cintia, Monte S. Angelo, 80126 Naples, Italy; ^3^ Scuola Superiore Meridionale, Largo S. Marcellino 10, 80138 Naples, Italy

**Keywords:** control theory, microbial consortia, cell populations

## Abstract

We address the problem of regulating and keeping at a desired balance the relative numbers between cells exhibiting a different phenotype within a monostrain microbial consortium. We propose a strategy based on the use of external control inputs, assuming each cell in the community is endowed with a reversible, bistable memory mechanism. Specifically, we provide a general analytical framework to guide the design of external feedback control strategies aimed at balancing the ratio between cells whose memory is stabilized at either one of two equilibria associated with different cell phenotypes. We demonstrate the stability and robustness properties of the control laws proposed and validate them *in silico*, implementing the memory element via a genetic toggle-switch. The proposed control framework may be used to allow long-term coexistence of different populations, with both industrial and biotechnological applications. As a representative example, we consider the realistic agent-based implementation of our control strategy to enable cooperative bioproduction of a dimer in a monostrain microbial consortium.

## 1. Introduction

Synthetic biology aims at engineering biological systems with new functionalities [1], with applications ranging from health treatments to bioremediation [2] and the production of biofuels and drugs in bioreactors [3]. This is made possible by embedding artificial genetic circuits into living cells, such as bacteria, yeast and fungi, modifying their natural behaviour [4]; that is, by synthetically modifying when and how much genes are expressed to produce proteins or other chemicals of interest. However, the level of complexity and the functions of such engineered genetic circuits are limited by intrinsic factors in the host cells, such as excessive metabolic burden, competition of limited resources and incompatible chemical reactions [5]. To overcome these limitations, a promising strategy is to distribute the required functionalities among multiple cell populations, forming a microbial consortium, so that each cell strain embeds a smaller subset of engineered gene networks [6–9]. In this way, each cell population carries out a specialized function and, by dividing labour with the others in the consortium, contributes more efficiently to the achievement of the overall final goal.

**Figure 1.**
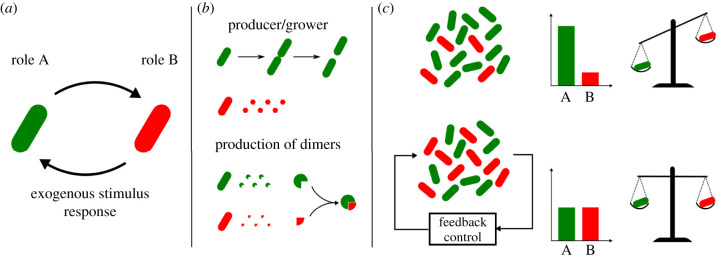
Microbial consortia composed of reversible differentiable cells can be balanced in real time by means of external feedback controllers to guarantee efficient labour division. (*a*) Reversible differentiable cells can carry out different roles by activating/deactivating specific sets of genes, depending on which state of the internal bistable memory is currently active. Cells can change role in response to exogenous stimuli from the environment, e.g. injection of inducer molecules or light. (*b*) Cells can, for example, either grow and duplicate or produce some desired molecule (red hexagons), or they can produce two different molecules that react and produce the desired final bioproduct (green and red circular sectors). (*c*) Cells expressing different genes also grow at different rates, and thus their coexistence can be compromised. Feedback control algorithms can be employed to regulate in real time the relative number of cells in the two groups, so that a balance in the population numbers and in the expression of desired genes is always guaranteed.

Unfortunately, this solution introduces an additional challenge: cells expressing different genes might also grow and divide at different rates. In particular, cells in the consortium whose function is associated with a lower metabolic burden will grow faster, eventually becoming dominant over the other populations, thus compromising the overall function of the consortium and giving rise to undesired spatio-temporal dynamics, such as oscillations or even extinction [10,11]. Therefore, it is crucial to develop methods to guarantee the stable coexistence between cell populations in a consortium by regulating and maintaining their relative numbers to some desired level, adjusting it to the requirements of the specific application of interest. This is possible, as suggested in [12], by using *feedback control algorithms* able to sense the relative size of all the populations involved and respond by applying appropriate stimuli to the cells in order to regulate their relative numbers. We proposed giving this problem the term *ratiometric control* of cell populations in [12] as its overall goal is to achieve and maintain a certain desired ratio between the size of the populations in the consortium, despite differences in their growth rates, noise and perturbations (figure 1*c*). Examples of external stimuli that can be applied to this aim include changes in the concentration of some inducer molecules in the growth medium or light stimuli applied via optogenetics.

Different solutions to regulate the relative size of coexisting cell populations have been proposed in the literature, mostly based on embedding additional genetic circuitry in the cells that make them able to sense and respond to each other's relative size [13–18]. Specifically, by sensing the density of the other group, cells can either increase their growth rate by producing some growth regulator protein (e.g. as in the Ecolibrium project, iGEM 2016 [19]) or decrease their number by means of toxin–antitoxin mechanisms (e.g. as in [13]). Unfortunately, these embedded solutions cannot avoid the possible extinction of one of the two species, which causes either uncontrolled growth of the survivor species or its death, and they are not flexible because the desired steady-state ratio of the two cell populations is hard-coded into the gene regulatory networks designed *ad hoc* and cannot be changed online. Moreover, in industrial applications where high efficiency is required, external control strategies [20–22] could be preferred to more sophisticated embedded solutions, because additional synthetic genes in the cells can cause lower production rates of the desired chemicals owing to the excessive metabolic load on the cells. Indeed, embedded controllers require the entire feedback loop to be engineered inside a single host; see, for example, [23]. That is, in addition to sensing and actuation mechanisms, they require the implementation of a signal-processing pathway able to link, according to some designed control policy, the outputs coming from the process to the actuation inputs able to modify the state of the process and also a comparator module to compare the process output with the reference signal.

In this paper, to solve this problem, we consider a microbial consortium composed of *reversible differentiable cells* [24], that is, cells that belong to the same strain and embed a genetic mechanism allowing them to keep memory of past states and adapt their behaviour to external stimuli from the environment, for example by activating/deactivating a specific set of genes. Specifically, we consider here the simplest case of cells that can switch between two states, mimicking a flip-flop or binary memory element (figure 1*a*). The state of this bistable memory encodes the current role played by the cells in the consortium, and therefore the set of genes they are expressing at that moment. For example, a cell can use its resources either to produce some molecule or to grow and divide, sustaining the cell population number [9] (figure 1*b*, top panel). Also, in the case of a genetic pathway divided into two parts, a cell can switch from one state to the other so as to activate either depending on the overall production levels in the consortium (figure 1*b*, bottom panel). We find that, by endowing the cell population with a reversible bistable system, an external control strategy can be used to solve the ratiometric control problem. Specifically, by applying external stimuli to all the cells in the consortium, it is possible to switch some cells from one state to the other so as to maintain the desired ratio. We show that this is possible in a number of different ways (namely, by using relay and proportional–integral (PI) controllers) and provide stability analysis of the resulting closed-loop system and exhaustive *in silico* validation of its performance and robustness. The validation is conducted by means of agent-based simulations in BSim [25,26], a powerful platform for realistic *in silico* experiments in bacterial populations. As a representative example, we consider the realistic agent-based implementation of the proposed ratiometric control strategy to enable cooperative bioproduction in microbial consortia, showing its effectiveness and flexibility when cell growth, cell-to-cell variability and other effects are appropriately modelled.

Although different approaches have been used to address the ratiometric control problem, their results hold under different sets of assumptions from those we use here. For example, in [21], the authors considered a microbial consortium already comprising two different cell strains, adjusting the dilution rate in a chemostat to regulate the relative numbers of the populations in the consortium, while the platform developed in [27], although powerful, relies on delivering a different control input to each cell and, in addition, all controlled cells are cultured in spatially distinct environments. Moreover, in [28], a non-reversible, efficient differentiation control mechanism has been proposed for the creation and maintenance of cellular sub-populations in single-strain microbial consortia, while a computer-controlled optogenetic platform for the regulation of the ratio of a two-strain *Escherichia coli* community has been recently presented in [29].

## 2. Results

### 2.1. Reversible differentiable cells can be controlled to a desired state via a common exogenous input

The cells we consider here are assumed to embed some mechanism that can store the memory of past events. In particular, we suppose that cells can be switched between two different states by appropriate external stimuli. The simplest model having this *memory-like property* (see §3.1 for further details) can be described by the following ordinary differential equation:

2.1 $${\dot{x}}_{i}=\eta_{i}x_{i}-x_{i}^{3}+u,$$

where u∈U⊂R is an input signal and is *common* to all cells.

**Figure 2.**
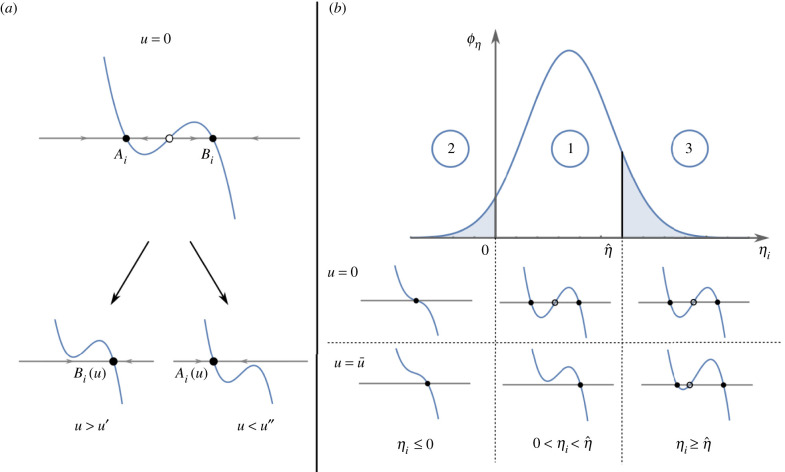
Reversible cells can be switched from state *A* to *B*, and vice versa, by means of a common external input. (*a*) The scalar dynamical system ${\dot{x}}_{i}=\eta_{i}x_{i}-x_{i}^{3}+u$, with *η*_*i*_ > 0, has two stable equilibrium points for *u* = 0, namely *A*_*i*_ and *B*_*i*_, each one corresponding to one of the two possible *roles* the cell can play in the consortium. A cell is *controllable* if, by varying the input *u* in its interval of definition U, it can be moved from one group to the other and vice versa. That is, there exists an admissible value $u^{\prime }\in U$ ($u^{\prime \prime }\in U$) such that there is a unique positive (negative) stable solution to the equation ${\dot{x}}_{i}=0$ when *u* > *u*′ (*u* < *u*″). Full and empty dots represent stable and saddle equilibria, respectively. (*b*) Not all cells might respond as desired owing to their heterogeneity, captured here by different values of the parameter *η*_*i*_ (assumed to be drawn from some probability distribution with density function $\phi_{\eta }$, here sketched as Gaussian, just for the sake of illustration). Only cells whose value of the parameter *η*_*i*_ is between 0 and $\hat{\eta }$ are *controllable* (case 1), that is, they have two stable equilibria for *u* = 0 and a unique stable equilibrium for $u=\pm \bar{u}$. Other cells can either be memory-less or *monostable*, that is, they have only one equilibrium point for all values of *u* (case 2) or they can be *unswitchable*, having two stable equilibria for every u∈U, and therefore cannot change role in the consortium (case 3).

In equation (2.1), $x_{i}\in X\subset R$ represents the macroscopic state, or role, of cell *i* and the value of parameter $\eta_{i}\in R$ is assumed to be different for all cells, accounting for their heterogeneous responses to the common external input signal *u*. For positive values of *η*_*i*_, the equation ${\dot{x}}_{i}=0$ with *u* = 0 has two stable, non-trivial solutions, one negative and another positive, that we denote as *A*_*i*_ and *B*_*i*_, respectively (figure 2*a*). These solutions are the stable equilibrium points of the dynamical system described in (2.1) when no input is applied. We define as $R_{A_{i}}=\{x_{i}\;:\;x_{i}<0\}$ and $R_{B_{i}}=\{x_{i}\;:\;x_{i}>0\}$ the regions of attraction ([30], Sec. 8.2) of *A*_*i*_ and *B*_*i*_, respectively. Each cell will asymptotically converge to either *A*_*i*_ or *B*_*i*_ depending on which region of attraction its initial condition belongs to. Moreover, we denote by $N_{t}$ the finite set of all cells in the consortium at time *t* and with its cardinality, that is, the number of cells currently under observation (e.g. via a fluorescence microscope). Note that this number may vary in time as a consequence of cell growth or death, or because of their removal (e.g. flush away) from the culture chamber in which they are hosted. We define the sets $A_{t}\;:=\{i\in N_{t}\;:\;x_{i}(t)\in R_{A_{i}}\}$ and $B_{t}\;:=\{i\in N_{t}\;\;:\;\;x_{i}(t)\in R_{B_{i}}\}$, such that $A_{t}\cup B_{t}=N_{t}$ and $A_{t}\cap B_{t}=\text{Ø}$, and denote with *n*_*A*_(*t*) and *n*_*B*_(*t*) their cardinality. These two sets represent the group of cells in the consortium that, at time instant *t*, in the absence of any control input *u* are expected to asymptotically converge to *A*_*i*_ and *B*_*i*_, respectively. Note that, as $A_{t}$ and $B_{t}$ form a partition of , at any time it holds that *n*_*A*_(*t*) + *n*_*B*_(*t*) = *N*(*t*).

We model cell-to-cell variability by assuming that the parameter *η*_*i*_ in (2.1) is drawn randomly for each cell from the real interval $\left[\underline{\eta },\;\bar{\eta }\right]$ with some probability distribution (figure 2*b*). Also, we assume that the magnitude of the control input *u* is upper bounded by some maximum value $\bar{u}\;:=\max_{u\in U}|u|$. In the presence of such a bound on the control signal, only cells whose parameter value *η*_*i*_ is smaller than the threshold value $\hat{\eta }$, defined as

2.2 $$\hat{\eta }=\sqrt[{3}]{\frac{27\;{\bar{u}}^{2}}{4}},$$

can be switched from one state to the other by an admissible value of *u*, that is, $u\in [-\bar{u},\bar{u}]$. We define cells fulfilling the condition $\eta_{i}\in (0,\hat{\eta })$ as controllable cells. For details on the derivation of $\hat{\eta }$, see Section S1 in the electronic supplementary material.

All cells not fulfilling this condition are instead uncontrollable because they are either *monostable*—their parameters differ so much from their nominal values that their bistable nature is lost—or *unswitchable* because their parameters exceed the threshold value (2.2). In the former case, *η*_*i*_ will be taking non-positive values in our model, that is, *η*_*i*_ ≤ 0, while in the latter case $\eta_{i}\geq \hat{\eta }$.

Therefore, all cells characterized by parameter values $\eta_{i}\in (0,\hat{\eta })$ can be switched from one state to the other by means of a common bounded external input *u* applied into the environment. It is therefore possible to design, for such a subset of controllable cells, some feedback control law to automatically regulate their state and keep the balance in the consortium between the two groups $A_{t}$ and $B_{t}$ to some desired level. As we are going to show next, uncontrollable cells will contribute to a small residual error that can be precisely estimated as a function of the upper bound $\bar{u}$ on the control input and therefore appropriately taken into account in applications.

Note that, even if model (2.1) is not a precise model of any existing bistable memory element, it captures its essential bistable nature and can therefore provide valuable information for the design of reliable and robust controllers able to solve the ratiometric control problem, as we show in the rest of this work.

### 2.2. Ratiometric control of cell populations can be achieved by using external feedback strategies

The goal of ratiometric control is to regulate and maintain the *relative ratios* between the number of cells in $A_{t}$ and the number of cells in $B_{t}$, defined as

2.3 $$r_{A}(t)=\frac{n_{A}(t)}{N(t)}\;\mathrm{and}\;r_{B}(t)=\frac{n_{B}(t)}{N(t)}.$$

As, by definition, *r*_*A*_(*t*) + *r*_*B*_(*t*) = 1 for all time, it suffices to control either *r*_*A*_ or *r*_*B*_ to control the other. Without loss of generality, we assume the ratio *r*(*t*) to be controlled is *r*_*B*_(*t*). Note that accurate measurement of each cell state is not needed. Indeed, if only noisy measurement of the state of the cell is available, as long as it is possible to identify the region of attraction the cell currently is in, the values of both *r*_*A*_ and *r*_*B*_ can still be accurately quantified.

More formally, the objective of the *ratiometric control problem* can be stated as follows.

**Objective.**

Given a consortium of reversible differentiable cells whose macroscopic dynamics can be described by (2.1) and a desired ratio *r*_d_ ∈ [0, 1], design a feedback control law *u* = *u*(*t*, *x*), where $x={\left[x_{1},\ldots ,x_{N(t)}\right]}^{⊤}$, such that at steady state the consortium is divided into two cell groups whose ratio converges to some desired value, *r*_d_, that is,

2.4 $$r(t)\to r_{d}\;\text{as}\;t\to \infty .$$

The previous statement can also be reformulated in terms of the control error signal *e*(*t*) := *r*_d_ − *r*(*t*), by requiring that it goes to zero at steady state, that is, *e*_∞_ = 0, where *e*_∞_ := lim _*t*→∞_ *e*(*t*). The definition of the control error does not guarantee *per se* that the cells express the desired phenotype, as the cells whose state are near the unstable equilibrium will not be producing the target compound at the desired rate. However, cells belonging to the correct region of attraction will converge, after some transient, to the correct equilibrium, where the desired phenotype is expressed.

Note that it is possible to guarantee the solution of the ratiometric control problem as defined in (2.4) for *any* *r*_d_ ∈ [0, 1] only if *all* cells in the consortium are controllable, as described in §2.1. Indeed, if part of the population is not controllable, a residual steady-state error might still be present (see §2.3 for details and an analytical estimate of such a residual error).

**Figure 3.**
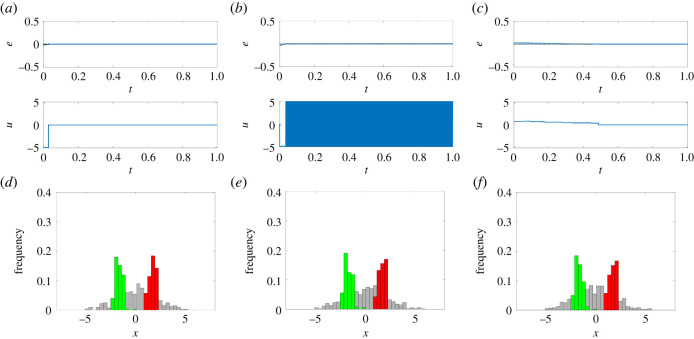
Feedback control strategies are effective to balance two groups of controllable reversible cells to a 1 : 1 ratio (*r*_d_ = 0.5). (*a*–*c*) Evolution of the error signal *e*(*t*) and of the control input *u*(*t*) for (*a*) the first implementation of the relay controller (3.5), (*b*) the second implementation of the relay controller (3.7), (*c*) the PI controller (3.9)–(3.10). (*d*–*f*) Distribution of the cells' state at the beginning of the simulation (*t* = 0 a.u., grey histogram) and at steady state (*t* = 1 a.u., green and red histograms), for (*d*) the first implementation of the relay controller, (*e*) the second implementation of the relay controller, (*f*) the PI controller. The green and red bars in (*d–f*) correspond to cells being in the region of attraction of *A*_*i*_ and *B*_*i*_, respectively. The maximum control input is set to $\bar{u}=5$ and the gains of the PI controller are set to *k*_P_ = 30 and *k*_I_ = 50. All cells (*N* = 400) have initial conditions *x*_*i*_(0) drawn from the normal random distribution N(0,4), and the parameters *η*_*i*_ are drawn with uniform distribution from the interval [1, 5]; therefore, all cells are controllable, as no monostable (*η* > 0) and no unswitchable ($\bar{\eta }<\hat{\eta }\approx 5.53$) cells are present in the population. (See also electronic supplementary material, figure S1 for more simulations with different desired ratios *r*_d_.)

We present here two different feedback control strategies to solve the ratiometric control problem (figure 3), an on–off relay controller and a PI controller. Both solutions are easy to implement, are robust and are often used in other control applications of cell populations in microfluidic devices [31–34] (see §3.3).

*Relay controllers* (also known as bang-bang controllers) are simple yet effective feedback control laws that, by comparing the current output of the process of interest with its desired value, generate a piece-wise constant input signal *u*_r_(*t*) whose value belongs to a discrete set $U_{r}$. Here, we propose the use of two alternative implementations of the relay controller, the former where the control input can also be set to zero, i.e. $U_{r}^{\prime }=\{0,\bar{u},-\bar{u}\}$, and the latter where *u*_r_ is always non-zero, i.e. $U_{r}^{\prime }=\{\bar{u},-\bar{u}\}$.

In the ideal case where all cells are controllable, the first implementation of the relay controller guarantees finite time convergence to zero of the error signal (figure 3*a*). The second implementation instead can only guarantee bounded convergence of the error to zero since the input signal cannot be turned off once the error reaches zero. Hence, when such an implementation is adopted, the control input will continue to oscillate between its possible values (figure 3*b*). As is common practice in applications where noise and uncertainties are unavoidable, a dead-zone or a delay can be added in the control loop to avoid high-frequency oscillations of the control input that may cause excessive stress to cells and to the actuation system [35]. The details of the proof of convergence for the proposed relay controllers are reported in Section S2 in the electronic supplementary material.

An alternative strategy is the use of a *PI controller* that generates a control input *u*_PI_(*t*) computed as the sum of one term that is proportional (P) to the error *e*(*t*) and another that is proportional to its integral (I) in time. In general, PI controllers guarantee zero regulation error at steady state in the presence of constant output disturbances [36]. In our implementation, this controller is complemented with a (anti-wind-up) reset condition that sets to zero the internal state of the integrator whenever the error signal *e*(*t*) is equal to 0 or changes its sign (see §3.3). When all cells are controllable, this strategy was also proved to solve the ratiometric control problem and guarantee convergence of the error to zero (see Section S2 in the electronic supplementary material).

The evolution of the error signal *e*(*t*) under the action of the PI controller is reported in figure 3*c*. The error converges to zero as expected and the control input *u*_PI_(*t*) also settles to zero after a short transient, similarly to what is observed in the first implementation of the relay controller presented before.

Effective balancing of groups of reversible cells is also achieved by feedback control when the goal is to achieve groups of different sizes, that is, for *r*_d_ different from 0.5, e.g. equal to 0.75 or 0.25, corresponding to the ratios 1 : 3 and 3 : 1, respectively (electronic supplementary material, figure S1). Note that the similarities of the plots for the relay and PI control actions in electronic supplementary material, figure S1 are mainly due to the different set points chosen in these simulations. Indeed, since the initial control error is bigger with respect to the case presented in the main text, the time needed for the integral control action to saturate the control input is relatively low. When this happens, the control actions generated by the relay and the PI controllers are identical.

The performance of the controller is also compared with open-loop simulations where no control is applied (electronic supplementary material, figure S7). While using a closed-loop control algorithm, it is possible to regulate the relative numbers at some desired value, independently of the initial conditions; in the absence of any control action, the error does not change over time and the final configuration of the consortium will strongly depend on the initial conditions chosen for the cellular population.

### 2.3. Robust bounded regulation of the ratio is still possible in the presence of cell variability and physical constraints

**Figure 4.**
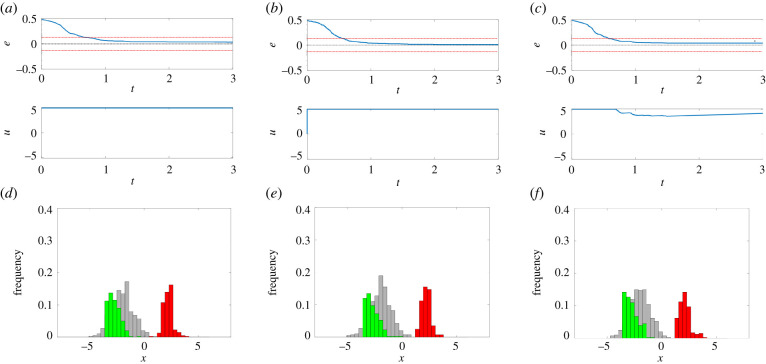
Balance to a 1 : 1 ratio is achieved with a residual steady-state error in the presence of uncontrollable cells (*r*_d_ = 0.5). (*a*–*c*) Evolution of the error signal *e*(*t*) and of the control input *u*(*t*) for (*a*) the first implementation of the relay controller (3.5), (*b*) the second implementation of the relay controller (3.7), (*c*) the PI controller (3.9)–(3.10). (*d*–*f*) Distribution of the cells’ state at the beginning of the simulation (*t* = 0 a.u., grey histogram) and at steady state (*t* = 3.0 a.u., green and red histograms), for (*d*) the first implementation of the relay controller, (*e*) the second implementation of the relay controller, (*f*) the PI controller. The green and red bars in (*d–f*) correspond to cells being in the region of attraction of *A*_*i*_ and *B*_*i*_, respectively. The maximum control input is set to $\bar{u}=5$ and the gains of the PI controller are set to *k*_P_ = 30 and *k*_I_ = 10. All cells (*N* = 400) have initial conditions *x*_*i*_(0) drawn from the normal random distribution N(−2,1), and the parameters *η*_*i*_ are drawn with uniform distribution from the interval [ −1, 14]; therefore, both monostable (*η* < 0) and unswitchable ($\bar{\eta }>\hat{\eta }\approx 5.53$) cells can be present in the population. The steady-state errors observed in the *in silico* experiment are equal to (*a*) 0.05, (*b*) 0.0225 and (*c*) 0.0825. Note that all the observed errors are below the theoretical upper bound on the control error that can be estimated using (2.5) as $e_{r}=e_{r}^{0}+e_{r}^{u}\approx 0.07+0.06=0.13$ (depicted in (*a*–*c*) as red dashed lines). (See also electronic supplementary material, figure S2 for more simulations with different desired ratios *r*_d_.)

When uncontrollable cells are present in the consortium, that is, cells that cannot be moved from one group to the other in response to any admissible inputs, the ratiometric control problem cannot be solved asymptotically, that is, we cannot guarantee that *e*_∞_ → 0 for an arbitrary initial configuration of the consortium. However, we can still guarantee that the absolute value of the steady-state error |*e*_∞_| will be upper bounded by some positive quantity *e*_*r*_, that is, |*e*_∞_| ≤ *e*_*r*_. This effect is well illustrated in figure 4, where it is shown that, regardless of the control algorithm being used, the error *e*(*t*) approaches, but does not converge exactly to, zero. The error bound at steady state will depend upon the interplay between heterogeneity of the cells' dynamics and the constraints on the maximum input value of $\bar{u}$ that can be applied to the cells, as discussed in §2.1.

The upper bound *e*_*r*_ can be estimated as being composed of two terms, that is,

2.5 $$e_{r}=e_{r}^{0}+e_{r}^{u},$$

each related to the probability of finding one of the two types of uncontrollable cells (i.e. monostable and unswitchable, respectively) in the consortium (figure 2). The first term, denoted as $e_{r}^{0}$, is related to the fraction of monostable cells, associated with a non-positive value of *η*_*i*_, and so admitting only one stable equilibrium point for all values of *u*. For *N* → ∞, where *N* is the number of cells in the population, assuming that the probability distribution from where the parameters *η*_*i*_ are drawn is known, we can estimate $e_{r}^{0}$ as

2.6 $$e_{r}^{0}=\mathrm{Pr}[\eta_{i}\leq 0],$$

where Pr denotes the probability measure. The second term, denoted as $e_{r}^{u}$, is related to the fraction of unswitchable cells, that is, cells that are bistable but cannot be switched by any admissible value of the control input u∈U. For a given upper bound input value $\bar{u}$, the fraction of unswitchable cells can be estimated, for *N* → ∞, as the probability that the parameter *η*_*i*_ is greater than , that is, $\mathrm{Pr}[\eta_{i}>\hat{\eta }]$. Therefore, the residual error at steady state owing to this second type of uncontrollable cells can be quantified as

2.7 $$e_{r}^{u}=\max\{0,\;\mathrm{Pr}[\eta_{i}>\hat{\eta }]-r_{d},\;\mathrm{Pr}[\eta_{i}>\hat{\eta }]-(1-r_{d})\}.$$

Equation (2.7) is derived by assuming that all the unswitchable cells are in the wrong region of attraction at the beginning of the simulation, which represents the worst-case scenario. Specifically, if either $\mathrm{Pr}[\eta_{i}>\hat{\eta }]-r_{d}>0$ or $\mathrm{Pr}[\eta_{i}>\hat{\eta }]\;-$ $(1-r_{d})>0$, it means that the amount of cells that need to be switched to achieve the control goal is greater than the number of switchable cells in the consortium, meaning that there may be some non-zero steady-state error. Note that this value depends on the relationship between the desired ratio for that group (either *r*_d_ or 1 − *r*_d_) and the number of unswitchable cells therein, because they affect the error only when this number exceeds the desired value (see electronic supplementary material, S3 for details).

Similar results are observed also when the desired goal is to split the cell population into groups of different sizes, e.g. with 3 : 1 or 1 : 3 ratios (electronic supplementary material, figure S2), confirming that this undesired effect is not due to the particular control strategy adopted or to the chosen desired ratio.

### 2.4. Ratiometric control enables cooperative bioproduction in microbial consortia

So far the analysis has been conducted by considering the scalar model (2.1) capturing the macroscopic bistable nature of the cells considered in this paper. As we are going to show by means of the representative application that follows, the behaviour captured by the reduced model in (2.1) is also qualitatively preserved in more complex and realistic cell models exhibiting the required memory-like property. Therefore, albeit simple, we demonstrate that the model in (2.1) can be effectively used to design feedback control laws to solve the ratiometric control problem in realistic applications.

**Figure 5.**
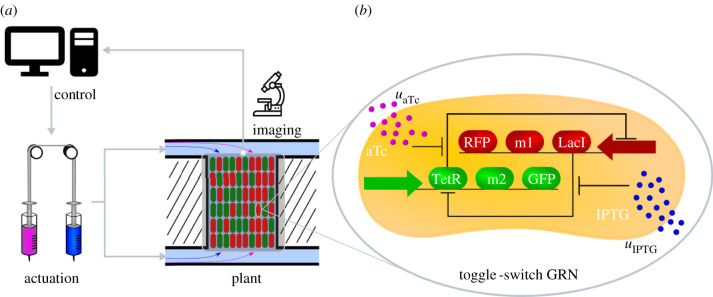
Controlled cooperative bioproduction of a dimer in microfluidic devices. (*a*) Reversible differentiable cells are hosted in microfluidic chambers, where they grow and produce a specific molecule related to the corresponding active state of their internal memory. The current ratio of the two cell groups in the chamber, and hence the production level of the corresponding monomers, is evaluated by measuring with a fluorescence microscope the expression of reporter proteins in each cell. This information is acquired by the feedback control algorithm that compares the current ratio *r*(*t*) with the desired ratio *r*_d_ and computes online the corresponding control inputs. Finally, these signals are sent to the cells by actuating a pair of syringes connected to the microfluidic chambers and containing mixtures of growth medium and inducer molecules. (*b*) The required reversible bistable memory mechanism is implemented by using an inducible toggle-switch. Depending on which of the two repressor proteins, either LacI or TetR, is currently expressed, the cell produces the corresponding monomer and the reporter protein (either M1 and red fluorescent protein (RFP) or M2 and green fluorescent protein (GFP), respectively). The state of the toggle-switch can be flipped by changing the concentration of the inducer molecules anhydrotetracycline (aTc) and isopropyl β-D-1-thiogalactopyranoside (IPTG) in the microfluidic chamber (denoted as *u*_aTc_ and *u*_IPTG_), which diffuse through the cell membrane and bind to TetR and LacI, respectively. GRN, gene regulatory network.

As a representative case of study, we consider the agent-based *in silico* implementation of ratiometric control for the bioproduction of protein dimers in microfluidic devices (figure 5). In this scenario, according to its state (*A* or *B*), each cell in the consortium produces either one of two monomers. By acting on the available control inputs, we want to regulate the relative number of cells producing the two monomers so as to balance the overall production of the resulting dimer.

We assume that the mechanism required by the *E. coli* cells to guarantee their correct coordinated behaviour is implemented by means of an inducible genetic toggle-switch [37]. Specifically, we consider the circuit design presented in [33] and further analysed in [38–41]. This genetic regulatory network consists of two repressor proteins, LacI and TetR, both repressing each other’s promoter, so that only one protein is fully expressed at any time. The expression level of the two repressor proteins can be flipped by changing the concentration of two inducer molecules, aTc and IPTG. The former input, aTc, binds to TetR, increasing the rate of production of LacI, and therefore causing the cell to converge to the steady state corresponding to high expression of LacI. Analogously, IPTG binds to LacI, causing the commitment of the cell to a steady state corresponding to high expression of TetR.

The sixth-order dynamical model of each cell is described in detail in Section S5 in the electronic supplementary material, in which the variables *u*_aTc_ and *u*_IPTG_ (as reported in figure 5) denote the concentrations of the inducer molecules in the growth medium of the microfluidic chambers and they represent the control inputs that can be applied to all cells to change their production role in the consortium.

We further assume that the genes *m1* and *m2* encoding the two monomers of interest are each transcribed together with the repressor genes *lacI* and *tetR* of the toggle-switch circuit. So that, at steady state, each cell fully produces only one monomer at the time and at a rate assumed to be proportional to the concentration of the corresponding repressor protein. Reporter genes of red and green fluorescent proteins (RFP and GFP) are also bound to the repressor genes to monitor the current level of production of the monomers by using fluorescence microscopy (figure 5). Finally, we assume that the two monomers have equal transcription and translation rates. Therefore, for the dimer to be produced at high rate, the consortium must be split and maintained into two symmetric groups with a 1 : 1 ratio, that is, we set *r*_d_ = 0.5. Note that this assumption does not hinder the generality of the framework presented, as different transcription and translation rates would simply require the consortium to operate around a different setpoint that we can reach and stabilize by just modifying *r*_d_.

In the *in silico* experiments, we also take into account realistic physical and technological constraints of a possible implementation in the microfluidic experimental platform described in [12,31]. The choice of such a platform derives from its extensive use in the context of external control [27,34,35]. Specifically, we consider constraints on (i) the possible classes of input signals that can be generated by the actuators, (ii) an upper bound on the switching frequency of the inputs to limit osmotic stress to the cells, (iii) a time delay accounting for the time the chemical inducers take to flow from the reservoirs to the cell chambers, and (iv) a safety lower bound on the sampling time of the measurements to avoid excessive photo-toxicity (see Section S6 in the electronic supplementary material).

*In silico* control experiments have been conducted by using *ad hoc* implementations in BSim [25,26] of the two feedback control algorithms presented in §2.2 (see Section S7 in the electronic supplementary material). To test the relay control strategy, we assumed that the actuation of the inputs is realized using an ordinary T-junction [42], which allows only one inducer species at a time to be injected into the microfluidic chambers. For the PI controller, we assumed that the actuation is realised by a dial-a-wave system, as described in [43]. This actuation system is more advanced than the previous one as it allows mixtures of the two inducers to be injected in different proportions into the chambers. Note that, although two inducers are needed to manoeuvre the state of the toggle-switch, they are constrained by our control strategy to be either mutually exclusive (for the relay controller) or in a convex combination (for the PI controller), and therefore they can be viewed as being a single input (see Section S7 in the electronic supplementary material). Both feedback control algorithms take into account the characteristics of the experimental platform, and in particular of the actuators. Full details about the control algorithms and the technological constraints of the platform are reported in Section S7 in the electronic supplementary material.

The agent-based simulations in BSim accurately capture the cells’ reproduction, the spatial distribution and geometry of the cells and of the microfluidic chambers, the diffusion of chemicals into the environment and, more importantly, flush-out of the cells from the chambers. Further details on the stochastic simulation algorithm, geometry and other parameters used for *in silico* experiments in BSim are reported in Section S6 in the electronic supplementary material.

**Figure 6.**
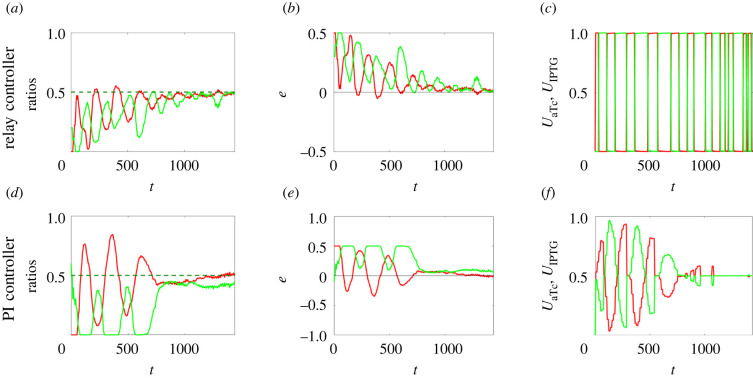
Cooperative production of two monomers to a 1 : 1 population ratio can be achieved by means of feedback ratiometric controllers in microfluidics. (*a,d*) Evolution in time of the populations’ ratios *r*_*A*_ (solid green line) and *r*_*B*_ (solid red line) with their respective desired reference values in dashed lines (*r*_d_ = 0.5), (*b,e*) of the error signals *e*_*A*_ (solid green line) and *e*_*B*_ (solid red line) and (*c,f*) inducer control signals *u*_aTc_ (solid red line) and *u*_IPTG_ (solid green line), normalized to their maximum values *U*_aTc_ and *U*_IPTG_, respectively. (*a–c*) Parameters of the relay control (equation (S30) in the electronic supplementary material): *U*_aTc_ = 60 ng ml^–^^1^; *U*_IPTG_ = 0.5 mM. (*d–f*) Parameters of the PI controller (equation (S32) in the electronic supplementary material): *U*_aTc_ = 100 ng ml^–1^; *U*_IPTG_ = 1 mM; *k*_P_, 1 = 100; *k*_P_, 2 = 1.5; *k*_I_, 1 = 1.5; *k*_I_, 2 = 0.05. Cells (about 200) in the simulated microfluidic chamber (with dimensions 40 μm × 50 μm × 1 μm) have the same parameter values, and their evolution has been obtained using the agent-based simulator BSim [25,26] (See Section S6 in the electronic supplementary material for further details on the simulator set-up). (See also electronic supplementary material, video S1 and figure S3 for more *in silico* experiments with different desired ratios *r*_d_.)

We observed that both controllers can successfully regulate, after relatively short transients, the populations’ ratio to the desired value (figure 6; electronic supplementary material), which otherwise would have converged to some value that strongly depended on the initial conditions of the cells (electronic supplementary material, figure S8). The relay controller shows a faster response with more severe oscillations (figure 6*a*–*c*), while the PI controller presents a smoother but slower response with higher accuracy at steady state (figure 6*d*–*f*). This is expected as it is well known that the relay control strategy is in general more robust to uncertainties and noise affecting the controlled process but has poorer accuracy at steady state; the PI control strategy shows better steady-state performance owing to the presence of an integral action.

The difference in the performance of the two strategies is also stressed by the different actuation systems employed in our experiments. Indeed, the dial-a-wave system allows for a finer regulation of the concentrations of the inducer molecules than the simpler (and cheaper) T-junction, allowing better accuracy of the control system at steady state. Similar performances are also obtained when the goal is changed to achieve different population ratios, e.g. a 1 : 3 ratio or a 3 : 1 ratio (electronic supplementary material, figure S3). These scenarios may correspond, for example, to situations in which the two monomers have equal transcription rates but different translation rates, requiring the consortium to be split into two asymmetric groups, for efficient production of the dimer.

Besides biochemical noise, the fluctuations at steady state (figure 6*a*,*d*) are essentially due to cells being flushed out of the microfluidic chamber as they grow and duplicate, and they do not depend on the control parameters used, which might only affect the transient response of the cell populations. These fluctuations are more relevant when cells are hosted in a small chamber and become less significant as the size of the growth chamber increases. Indeed, for the sake of simplicity, assuming the chamber to be a square of side ℓ, the magnitude ɛ of the fluctuations is proportional to 1/ℓ (see Section S4 in the electronic supplementary material); hence, the fluctuations increase as the chamber size decreases (see electronic supplementary material, figure S4 and video S2), and vice versa. In addition to this, we assessed the robustness of the control algorithms to cell-to-cell variability, modelled as variability between cell parameters. In detail, the numerical simulations confirmed that the ratiometric controllers presented here were also able to regulate the relative numbers in this case, as shown in electronic supplementary material, figure S5. The coefficient of variation (CV) was selected to be CV = 0.2, similarly to what has been done previously in the literature [7].

Another important factor determining the evolution of the relative numbers between sub-populations in microbial communities is their generally different growth rates (assumed so far to be identical for both phenotypes) owing to different metabolic loads. To test the robustness of the designed algorithms to this issue, we assumed that over-expression of the LacI pathway caused a reduction (of $(1-r_{d})>0$50%) of the growth rate. We found that, even when significant discrepancies in the growth rates are present, no divergence phenomena were observed, with the cells still splitting into two sub-populations with a steady-state error that never exceeds 0.15 (see electronic supplementary material, figure S6).

## 3. Methods

### 3.1. Memory-like property

We assume that the macroscopic behaviour of any cell in the consortium we wish to control can be modelled in the domain of interest by a dynamical system of the form

3.1 $$\dot{z}=g(z,w),$$

with being a smooth vector field, $z\in Z\subset R^{d}$ the state variables and $w\in W\subset R^{m}$ the exogenous input variables, representing, for instance, the concentrations of chemical species inside the cell and those of control inducer molecules into the environment, respectively. We assume that stochastic effects, such as fluctuations due to biochemical reactions, do not significantly alter the behaviour of the system at steady state; that is, the region of attraction of any stable equilibrium point of the dynamical system $\dot{z}=g(z,w)$ is large enough so that stochastic noise does not cause undesired switches from one equilibrium point to the other.

As discussed in the Introduction and in §2.1, we are interested in robustly regulating the behaviour of reversible cells; in particular, we focus our attention on a specific class of cells whose dynamics satisfy the following fundamental property.

**Definition.**

Consider a dynamical system of the form

3.2 $$\dot{z}=f\;(z,\alpha ),$$

where z∈Z and the parameter α∈I⊂R depends on some exogenous input signal w∈W, that is, *α* = *α*(*w*). We say that system (3.2) has a *memory-like property* if

— there exists some $\bar{\alpha }$ such that the system $\dot{z}=f\;(z,\bar{\alpha })$ has two stable equilibria and an unstable equilibrium; furthermore, the regions of attraction of the two stable equilibria form a partition of Z.
— there exist two values ${\hat{\alpha }}_{1}$ and ${\hat{\alpha }}_{2}$ such that for the system $\dot{z}=f\;(z,\bar{\alpha })$ has a single equilibrium point whose region of attraction is the whole Z.

### 3.2. Event-driven modelling of the control error evolution

Here, we derive an event-driven model for the evolution of the control error *e*(*t*) = *r*_d_ − *r*(*t*), presented in §2.2. Recall that the finite set of all cells in the consortium and its cardinality at time *t* are denoted by Z$N_{t}$ and *N*(*t*), respectively. Now, we denote with $E_{A\to B}^{i}(t^{\prime })$ the event at time instant *t* = *t*′, corresponding to when the state *x*_*i*_ of cell $i\in N_{t}$ enters the region of attraction of the equilibrium point *B*_*i*_, that is, for *t* ∈ [*t*′, *t*′ + ɛ] and $x_{i}(t)\notin R_{B_{i}}$ for *t* ∈ [*t*′ − ɛ, *t*′), where ɛ is a small positive real number. Likewise, we denote by $E_{B\to A}^{i}(t^{\prime \prime })$ the event at time instant *t* = *t*″, corresponding to when the state of cell $i\in N_{t}$ enters the region of attraction of *A*_*i*_. Specifically, for solutions to dynamical system (2.1) with *u* = 0 and *η*_*i*_ > 0, an event $E_{A\to B}^{i}$ occurs when the state of cell *i*, *x*_*i*_, crosses zero and becomes positive, while an event $E_{B\to A}^{i}$ occurs when *x*_*i*_ becomes negative. In this case, the threshold at zero is defined by the unstable equilibrium point at the origin dividing the regions of attraction of *A*_*i*_ and *B*_*i*_. Moreover, we denote by $E_{A\to B}$ ($E_{B\to A}$) the set of all events $E_{A\to B}^{i}$ ($E_{B\to A}^{i}$) occurring for all *i* at any time *t*, and we denote by E the set of all events occurring in the population, that is, $E\;:=E_{A\to B}\cup E_{B\to A}$.

To derive the discrete (event-driven) dynamics of the control error *e*(*t*), we make the following standing assumptions.

**Assumption 3.1.**

At any time *t* only one event in E can occur, that is, there exists a unique $i\in N_{t}$ such that either $E_{A\to B}^{i}$ or $E_{B\to A}^{i}$, but not both, occurs at time *t*.

**Assumption 3.2.**

The number of cells in the host chamber is assumed to be constant, that is, *N*(*t*) = *N*, for all *t*.

Assumption 3.1 implies that any two events in E cannot occur simultaneously. Assumption 3.2 follows from the fact that, after a short transient from the beginning of the experiment, cells grow, occupying the entire host chamber. From this time on, cells exceeding the maximum capacity of the chamber are flushed out. Therefore, the number of cells in the chamber can be assumed with a good approximation to be constant (except for a small, negligible oscillation due to flush-out, further discussed in Section S4 in the electronic supplementary material). Note that assumption 3.2 is true not only in the case of a finite-dimensional microfluidic device. Indeed, in industrial applications where chemostats are employed to maintain the density of the microbial culture to constant levels, it is reasonable to assume that the number of cells is also constant. However, in the case of large-scale cell co-cultures, such as when the experiments are run in bioreactors, since measuring the fluorescence levels of each individual in the consortium can become infeasible as the number of cells increases, the method we propose will need to be adapted, for example by measuring the average fluorescence in the whole population.

Under the assumptions above, there exists a sequence of discrete-time instants ${\left\{t_{k}\right\}}_{k\in N}$, each one corresponding to the occurrence of an event in E and such that *t*_*k*+1_ = *t*_*k*_ + Δ*t*_*k*_, where Δ*t*_*k*_ > 0 is the time interval between two consecutive events occurring at time *t*_*k*_ and *t*_*k*+1_, respectively. Moreover, from assumption 3.2, it follows that the functions *n*_*A*_(*t*) and *n*_*B*_(*t*), defining the number of cells converging to either *A*_*i*_ or *B*_*i*_, respectively, are piece-wise constant functions, that is, *n*_*A*_(*t*) = *n*_*A*_(*t*_*k*_) and *n*_*B*_(*t*) = *n*_*B*_(*t*_*k*_), $\forall t\in [t_{k},t_{k+1})$. Since *n*_*A*_(*t*) and *n*_*B*_(*t*) are constrained by the relation *n*_*A*_(*t*) + *n*_*B*_(*t*) = *N*, for all *t*, for the sake of brevity, we will refer to *n*(*t*) : = *n*_*B*_(*t*) only; *n*_*A*_(*t*) being given by *N* − *n*_*B*_(*t*).

That said, with *n*(*t*) being the number of cells in the region of attraction of *B*_*i*_ at time *t*, we can write the following discrete-time update law:

3.3 $$n(t_{k+1})=\left\{ \begin{array}{cc} n(t_{k})+1,\; & \text{if an event in}\;E_{A\to B}\;\text{occurs}\;\\ n(t_{k})-1,\; & \text{if an event in}\;E_{B\to A}\;\text{occurs.} \end{array}\right.$$

As a consequence, since $e(t)=r_{d}-\frac{n(t)}{N}$, we have that when an event in $E_{A\to B}$ occurs, then

$$e(t_{k+1})=r_{d}-\frac{n(t_{k+1})}{N}=\left(r_{d}-\frac{n(t_{k})}{N}\right)-\frac{1}{N}=e(t_{k})-\frac{1}{N}.$$

A similar reasoning holds when an event in $E_{B\to A}$ occurs. Therefore, the discrete-time dynamics of the control error can be expressed as

3.4 $$e(t_{k+1})=\left\{ \begin{array}{cc} e(t_{k})-\frac{1}{N},\; & \text{if an event in}\;E_{A\to B}\;\text{occurs}\;\\ e(t_{k})+\frac{1}{N},\; & \text{if an event in}\;E_{B\to A}\;\text{occurs.} \end{array}\right.$$

### 3.3. Design of the proposed feedback control algorithms and error dynamics

Here, we discuss the design of the control strategies proposed to solve the ratiometric control problem, namely the relay controller and the PI controller.

*Relay control algorithm.* A *relay controller* is a feedback control law that generates a piece-wise constant input *u*_r_(*t*) by comparing an output measured from the plant with some desired reference value *r*_d_. The input *u*_r_(*t*) takes its value from a finite set of real values $U_{r}$, generally composed of only two values, one chosen such that the control error *e*(*t*) = *r*_d_ − *r*(*t*) decreases when *e*(*t*) > 0 and the other such that it increases when *e*(*t*) < 0.

We have considered two implementations of the relay controller. Specifically, the first implementation includes a control shutdown condition when *e*(*t*) = 0, and the second one does not.

Formally, the *first* implementation of the relay control input is defined as

3.5 $$u_{r}^{\prime }(t)=\left\{ \begin{array}{cc} \bar{u},\; & e(t)>0\;\\ 0,\; & e(t)=0\;\\ -\bar{u},\; & e(t)<0. \end{array}\right.$$

The value $\bar{u}>0$ is chosen such that (ideally) all cells are controllable, that is, for $u_{r}^{\prime }=\bar{u}$ (), the equation $\eta_{i}x_{i}-x_{i}^{3}+u_{r}^{\prime }=0$ has a unique stable solution, namely ${\bar{x}}_{i}=B_{i}(\bar{u})$ (${\bar{x}}_{i}=A_{i}(\bar{u})$), for all *i* such that *η*_*i*_ > 0. This guarantees that, when *e*(*t*) > 0 (i.e. *r*_*B*_(*t*) < *r*_d_), the next event occurring must belong to $E_{A\to B}$, forcing the error to decrease according to (3.4), that is, *e*(*t*_*k*+1_) = *e*(*t*_*k*_) − (1/*N*). Likewise, when *e*(*t*) < 0 (i.e. *r*_*B*_(*t*) > *r*_d_), $u_{r}^{\prime }=-\bar{u}$ implies that the next event occurring belongs to $E_{B\to A}$, and so $e(t_{k+1})=e(t_{k})+\frac{1}{N}$. Moreover, note that, when no control is applied (i.e. *u*_r_′ = 0), each cell will converge to either *A*_*i*_ or *B*_*i*_, depending on its current state, without any other event in E having to occur. Therefore, the shutdown condition ensures that if there exists a t* such that *e*(t*) = 0, then *e*(*t*) = 0 for all *t* ≥ t*. Combining (3.5) and (3.4), the discrete-time update law for the control error becomes

3.6 $$e(t_{k+1})=\left\{ \begin{array}{cc} e(t_{k})-\frac{1}{N},\; & \text{if}\;e(t_{k})>0\;\\ e(t_{k}),\; & \text{if}\;e(t_{k})=0\;\\ e(t_{k})+\frac{1}{N},\; & \text{if}\;e(t_{k})<0. \end{array}\right.$$

The previous discrete map can also be rewritten as *e*(*t*_*k*+1_) = *e*(*t*_*k*_) − (1/*N*)sgn(*e*(*t*_*k*_)).

The *second* implementation of the relay control input, without the shutdown condition, is defined as

3.7 $$u_{r}^{\prime \prime }(t)=\left\{ \begin{array}{cc} \bar{u},\; & e(t)\geq 0\;\\ -\bar{u},\; & e(t)<0. \end{array}\right.$$

In this case, it is not possible to ensure that the error remains equal to zero indefinitely. By using a similar reasoning as before, (3.4) can be recast as

3.8 $$e(t_{k+1})=\left\{ \begin{array}{cc} e(t_{k})-\frac{1}{N},\; & \text{if}\;e(t_{k})>0\;\\ -\frac{1}{N},\; & \text{if}\;e(t_{k})=0\;\\ e(t_{k})+\frac{1}{N},\; & \text{if}\;e(t_{k})<0. \end{array}\right.$$

*PI control algorithm.* The control input of the PI controller is defined as

3.9 $$u_{\mathrm{PI}}(t)=k_{P}e(t)+k_{I}z(t)$$

and

3.10 $$\dot{z}(t)=e(t),\;z(0)=0,$$

with *k*_P_ and *k*_I_ being positive constants. This control action is complemented with a (anti-wind-up) reset condition that sets the internal state *z* of the integrator to zero whenever the error becomes 0 or changes its sign. Furthermore, to take into account constraints on the actuation system of the experimental platform, the control input signal *u*_PI_ is assumed to be saturated at $\bar{u}$ and $-\bar{u}$.

The control algorithm guarantees that when *e*(*t*) > 0 the control input *u*_PI_(*t*) is positive and $\frac{d}{dt}u_{\mathrm{PI}}(t)>0$. So, *u*_PI_(*t*) will increase and reach some positive value $\hat{u}$ such that, for at least one cell, the equation $\eta_{i}x_{i}-x_{i}^{3}+u=0$ with $u=\hat{u}$ and *η*_*i*_ > 0 has a unique solution, namely $B_{i}(\hat{u})$. The cell will be attracted by this stable equilibrium point and, therefore, there will exist a time instant *t*′ such that, for all *t* ≥ *t*′, and an event in $u_{\mathrm{PI}}(t)\geq \hat{u}$$E_{A\to B}$ will occur. A similar reasoning holds in the case *e*(*t*) < 0. Hence, it directly follows that the discrete-time update law for the control error *e*(*t*) under the PI control law is the same as in (3.6). The details of the proof of convergence for the proposed controllers are reported in Section S2 in the electronic supplementary material.

## 4. Discussion

We presented a general framework to guide the design of external feedback controllers for phenotype regulation in microbial consortia. We showed that, by exploiting the memory-like property of reversible differentiable cells, a single-strain cell population can be divided into two groups, expressing different sets of genes, whose relative numbers, i.e. the ratio, can be regulated by means of common exogenous inputs. We showed by means of a representative example that ratiometric feedback controllers can robustly stabilize a cell population, endowed with a genetic toggle-switch functioning as a bistable memory, and can guarantee the balance between the two groups of cells even in the presence of realistic physical and technological constraints of the experimental microfluidic platform we considered. Note that, although the proposed controllers can effectively regulate the ratio of the two sub-populations, it is not possible to regulate the rate of convergence at which cells express the desired level of the phenotype of interest, as this is determined by the inherent dynamics of the cells. Moreover, our *in silico* experiments highlighted that the control algorithms we presented can also shape the composition of the consortium in the presence of stochastic effects, despite the fact that we proved the convergence of the closed-loop system only in the deterministic scenario.

A fundamental open problem in multicellular control applications is to guarantee the coexistence of different microbial strains growing in the same environment. Although some solutions were proposed in the literature that rely on the use of additional genetic pathways embedded into the cells and non-reversible differentiation systems [28,44], the ratiometric control framework we presented here provides an alternative approach that might be more appropriate in other scenarios; for example, in industrial applications, where efficient production is strongly required. We wish to emphasize that the framework can be used as a guideline to design control strategies that are able to work at scales larger than microfluidics. For example, using a combination of flow cytometry, chemostats and optogenetics, such as the one developed in [44], it would be possible to culture such a cell population by embedding optogenetically inducible memory mechanisms and control their relative expression states at large scales.

Finally, we wish to highlight that ratiometric control of a population of reversible cells by means of a common input signal is only made possible by the heterogeneity of their response to that input. Indeed, heterogeneous reversible cells characterized by different parameter values switch at different time instants when subject to the same input, and this is a crucial property that allows their state to be controlled by an external feedback action. Stochastic effects, such as biochemical noise or delays, by amplifying the cell-to-cell variability can indeed facilitate the stabilization of the reversible cells into different groups, as was demonstrated in the *in silico* experiments we provided. A more in-depth analytical investigation of their effect is left for future work.

## Data Availability

The code used for all simulations and the electronic supplementary material, videos are available at https://github.com/diBernardoGroup/Ratiometric-Control-of-bacterial-populations. The data are provided in the electronic supplementary material [45].
